# Supplementary material for: Factors influencing treatment success in drug-susceptible tuberculosis patients in Ghana: A prospective cohort study
Source: PLOS Glob Public Health. 2025 Feb 21;5(2):e0004146. doi: 10.1371/journal.pgph.0004146 (PMC11844882; doi:10.1371/journal.pgph.0004146)
Supplement: Attachment — Submitted filename: Response to Reviewers.docx [file pgph.0004146.s003.docx]

Department of Pharmacotherapeutics and Pharmacy Practice,

School of Pharmacy and Pharmaceutical Sciences,

University of Cape Coast,

Cape Coast

Ghana

31^st^ December 2024

Academic Editor

PLOS Global Public Health

Subject: Rebuttal to Reviewer Comments on Manuscript Submission PGPH-D-24-02857

Dear Lei Gao,

We appreciate the opportunity to respond to the insightful comments provided by the academic editor and reviewers regarding our manuscript titled "Factors Influencing Treatment Success in Drug-Susceptible Tuberculosis Patients in Ghana: A Prospective Cohort Study." We have carefully considered each point raised and revised the manuscript accordingly. Below, we provide detailed, point-by-point responses to the concerns raised:

1. **Abbreviation of HRQOL**:
   - HRQOL has been rewritten in full at its first mention and abbreviated in brackets, as suggested by the reviewer (Line 95).
2. **Rewriting of results from lines 196–198** **and 211 - 218**
   - The content has been rewritten in the results section (191 – 203) and further discussed in the Discussion section (lines 280–289).
3. **Discussion of demographic factors and treatment success (line 139)**:
   - The influence of other demographic factors on treatment outcomes has been elaborated in the last paragraph of the Discussion section (lines 291–300).
4. **Discussion of factors associated with loss to follow-up**:
   - The discussion has been revised (lines 245–252) to state:

"This study did not find significant associations between TB treatment default rates and factors such as sex (p = 0.874), marital status (p = 0.190), educational level (p = 0.119), baseline physical health impairment (p = 0.243), baseline mental health impairment (p = 0.049), or age (p = 0.908). While previous studies [8,28] have reported that age, gender, and employment status influence TB treatment default rates, these associations were not observed in this study. This discrepancy may be attributed to the relatively low number of defaulters in the sampled population. Further research with larger sample sizes is needed to validate these findings and investigate these potential relationships more comprehensively."

**Additional Editor Comments:**

6. **Drug resistance status of TB patients**:
   - All patients sampled were drug-susceptible TB patients, confirmed via GeneXpert test routinely. This was an inclusion criterion for the study (Line 89-90).
7. **Omission of HIV status in the multivariate analysis**:
   - HIV status was excluded from the multivariate analysis in Table 4 due to collinearity, as its strong correlation with other variables could distort the analysis results.
8. **Update on References**

All references have been checked to be valid. Three new references have been cited in the manuscript. These include reference numbers 47 – 49.

We believe these revisions have strengthened the manuscript and fully addressed the concerns raised by the academic editor and reviewers. We sincerely thank the editorial team for their valuable feedback and thoughtful consideration of our submission.

Sincerely,

Richard Delali Agbeko Djochie, PhD

Tel: +233244101944

Email: [richarddjochie@gmail.com](mailto:richarddjochie@gmail.com/)

Alt email: [richard.djochie@ucc.edu.gh](mailto:richard.djochie@ucc.edu.gh)
