# Supplementary material for: Factors influencing treatment success in drug-susceptible tuberculosis patients in Ghana: A prospective cohort study
Source: PLOS Glob Public Health. 2025 Feb 21;5(2):e0004146. doi: 10.1371/journal.pgph.0004146 (PMC11844882; doi:10.1371/journal.pgph.0004146)
Supplement: Attachment — Submitted filename: Reviewer comments.docx [file pgph.0004146.s001.docx]

**Reviewer comments**

**Factors Influencing Treatment Success in Drug-Susceptible Tuberculosis Patients in**

**Ghana: A Prospective Cohort Study**

| **Line number** | **Line in the manuscript** | **Comment** |
| --- | --- | --- |
| 93 | To evaluate HRQOL, participants completed the Short-Form 12 version 2 (SF-12v2) survey | Please write HRQOL in full for the first in-text mention, abbreviate in brackets and then continue with the abbreviation. |
| 196-198 | Conversely, gender, alcohol use, tobacco smoking, and adverse drug reactions (ADRs) did not significantly affect treatment outcomes. Notably, treatment success rates for participants with and without ADRs were nearly identical, and the severity of ADRs did not influence outcomes. | This belongs to the **Discussion** section, comparing the results with those of other studies conducted in similar settings with almost similar socioeconomic statuses. |
| 211-218 | HIV status also played a critical role. HIV-negative participants had significantly greater odds of achieving treatment success compared to their HIV-positive counterparts. Among HIV-positive individuals, those with HIV-1 infection were substantially more likely to succeed compared to those with HIV-2 infection. Health-related quality of life at the start of treatment further influenced outcomes. Good baseline physical and mental health were both associated with increased odds of treatment success. Adverse drug reactions impacted treatment success in specific ways. Participants who did not experience gastrointestinal ADRs had higher odds of success, while those without hepatobiliary ADRs had markedly greater odds of achieving treatment success. | This section belongs to the **Discussion** as well, where findings are compared with those of other studies. The Results section should strictly present results without including the author's perceptions of the findings, as these are reserved for the Discussion section. While the author can elaborate on the results in this section, it should be a concise paragraph that directly reflects the data presented in the table, avoiding any interpretative commentary. |
| 139 | Of the 378 participants, most were male (67.2%), married (44.2%), and employed (75%), with an | While reading the Results section, I anticipated a discussion on the significance of marital status in relation to factors contributing to treatment success. However, this was not addressed in the Discussion section, even though stigma and mental health status were mentioned. Since marital status was included in the report form in REDCap and analyzed, it would be valuable to include a discussion on this variable. Different studies have explored the relationship between family structure, support, marital status, and treatment compliance. This could also be compared with treatment success versus lost to follow-up, discussing whether the authors' findings align with or contradict findings from other studies. |
| 158 | Adverse outcomes included 13.5% lost to follow-up… | The 13.5% lost to follow-up is highly significant and warrants *discussion* as it directly impacts poor anti-TB therapy outcomes. It would be beneficial to explore their marital status, employment status, and baseline HRQOL. Rather than focusing on individual factors, the discussion should emphasize commonalities among these individuals, providing insights into shared characteristics that might contribute to this outcome. This is just a suggestion. |

Overall, the manuscript is well written, with only minor adjustments needed. However, the study has collected rich data, and it may be worthwhile to discuss a broader range of the data collected.

-END-
